# Election of Professors, &c., by Concours

**Published:** 1847-10

**Authors:** 


*Election of Professors, &c., by Concours.*—We copy the following editorial article, from the Philadelphia Medical Examiner. We thank our friend, the able editor of that spirited Journal, for remembering us in any connection, but he is under a misapprehension in supposing, as he appears to do, that we have advocated the importation of the French system of *concours* into this country. We have never offered any remarks on that subject, but it has been our intention to do so for some time, and to express our dissent from the views entertained by some of our most respected brother

Journalists. The trial by *concours* of individual attainments, cannot, in our opinion, afford a guaranty that the successful competitor will become the best teacher. On the other hand, it must often happen that those who have the best qualifications for professorial duties, will be distanced in this preliminary race. Nor is this system secure against favoritism, and various extrinsic influences. The successful candidate having attained his end, is thereafter a fixture, not easily removed, if he fail to realize what was expected of him. This is an evil of no little account. We believe in all sincerity, that we have in this country, virtually, a system of *concours* as good as could be devised. If any one be disposed to teach, and believes himself to possess the capacity, attainments and industry which promise success, he will probably not find it a very difficult matter to place himself in a position which will afford some demonstration of his capabilities. If he manifest a decided fitness for the duties and responsibilities of public instruction, there is no danger that his talents will not be in demand. This follows as a necessary consequence of the principles upon which medical institutions are conducted in this country. The emoluments ot teachers depend on the prosperity of the schools with which they are connected; and this prosperity on the other hand, is dependent in a great measure, if not chiefly, upon the ability of its teachers. There is a mutual dependence, and the interests of both, and of those, too, who receive instruction, are reciprocal. Hence, while in the selection of Professors, the object paramount to all others is to secure the services of those most competent to teach, they must on their part derive strong incentives to constant exertion from the consciousness that both reputation and profit are dependent upon the degree of credit with which they discharge their duties. Should any one mistake his calling, and his efforts to teach acceptably prove a failure, there is a certain remedy in the obvious evidences of dissatisfaction which will not fail to attend him should he persist in warring against his destiny. The force of public sentiment in this conntry, in such cases, is far more available and summary than formal or legal proceedings. We venture the assertion that no public teacher in an American institution, can long maintain his connection with it, if he prove himself unfitted for the station. With these views we are not among those who think the system of *concours* would be an improvement upon the present condition of things here. We do not deny that there is need of reform in our educational institutions, and ample room for their improvement, but we are disposed to doubt if the schools of any country are superior to our own, except in greater facilities and resources for instruction. Many will doubtless think this is a rash assertion, but we firmly believe it is capable of being sustained. We may renew discussion of the topics upon which we have offered these few remarks, in a future number; in the mean time, we invite our readers to peruse the article of our contemporary already referred to:—

"Among other reforms recently advocated, the French practice of concouring for professorships has of late been highly commended by some of our brother journalists. In fact, we at one time advocated it ourselves, but afterward came to learn that its practical operation was not quite equal to its promises in theory. We understood from different sources, that instead of affording a true test, positive and relative, of the fitness of a candidate, it was liable to great abuses, and often led to deception: it was discovered, indeed, that many things were necessary fully to qualify a man to occupy a professor's chair beside those elicited by the concour. Our brethren of the Buffalo Medical Journal and of the St. Louis Medical and Surgical Journal, with many others, will be surprised to read the following, which we extract from the London Medical Gazette of the 16th of July,

'*Abolition of the Concours in France.*—The Chamber of Peers has come to a vote by which the system of election by Concours in France is abolished. Some of the noisy advocates of this electioneering practice are about to present a protest to the Chamber of Deputies against this vote, and to require a restoration of their favorite panacea for bringing out professional talent! But the felling of the most eminent and experienced men in the profession is decidedly against the re-establishment of this system.'

The introduction of this system into France some years since was hailed as the greatest improvement—as closing the door for ever upon favoritism, and opening the portals wide to genius and learning! And now it is discovered that common sense and business qualifications are quite as necessary as genius and great learning, and that these are not always combined in the same individual. *Change*, in fact, has not turned out to be *improvement.* Not only the professors of the colleges, but likewise the physicians and surgeons of the hospitals, in France, were chosen by concour, and consequently the strife was going on all the time. 'Thus,' says the Gazette, 'last year there were two vacancies for the situation of surgeons to the hospitals of Paris; *there were thirty-two candidates, and the concours lasted five months!*' Nothing could demonstrate the impracticability of such a system better than this fact.

